# Electrocardiogram Baseline Wander Suppression Based on the Combination of Morphological and Wavelet Transformation Based Filtering

**DOI:** 10.1155/2019/7196156

**Published:** 2019-03-03

**Authors:** Xiang-kui Wan, Haibo Wu, Fei Qiao, Feng-cong Li, Yan Li, Yue-wen Yan, Jia-xin Wei

**Affiliations:** ^1^Hubei Key Laboratory for High-efficiency Utilization of Solar Energy and Operation Control of Energy Storage System, Hubei University of Technology, Wuhan 430068, China; ^2^Hubei Collaborative Innovation Center for High-efficiency Utilization of Solar Energy, Hubei University of Technology, Wuhan 430068, China; ^3^Hubei Power Grid Intelligent Control and Equipment Engineering Technology Research Center, Wuhan 430068, China; ^4^Faculty of Health, Engineering and Sciences, University of Southern Queensland, Toowoomba, QLD 4350, Australia

## Abstract

One of the major noise components in electrocardiogram (ECG) is the baseline wander (BW). Effective methods for suppressing BW include the wavelet-based (WT) and the mathematical morphological filtering-based (MMF) algorithms. However, the *T* waveform distortions introduced by the WT and the rectangular/trapezoidal distortions introduced by MMF degrade the quality of the output signal. Hence, in this study, we introduce a method by combining the MMF and WT to overcome the shortcomings of both existing methods. To demonstrate the effectiveness of the proposed method, artificial ECG signals containing a clinical BW are used for numerical simulation, and we also create a realistic model of baseline wander to compare the proposed method with other state-of-the-art methods commonly used in the literature. The results show that the BW suppression effect of the proposed method is better than that of the others. Also, the new method is capable of preserving the outline of the BW and avoiding waveform distortions caused by the morphology filter, thereby obtaining an enhanced quality of ECG.

## 1. Introduction

Electrocardiogram (ECG) is an important clinical tool for heart disease diagnosis; hence, precision of ECG is a matter of life and death. However, the quality of the ECG signal is degraded during acquisition due to the interferences including power line harmonics, motion artifact, and baseline wander (BW), which makes it difficult to identify the factors which reflect the characteristics of physiological activity. As a consequence, interference suppression should be applied before the analysis of ECG [1]. Notably, the most important step is BW suppression which produces a stable signal for subsequent processing and for reliable visual interpretation.

BW embedded in ECG is mainly caused by the movement and respiration of the patient; consequently, it appears as low-frequency artifacts [2]. Unfortunately, although the high-pass filter is capable of suppressing BW, the ECG waveform distortion is inevitable because of the frequency variations of the ECG signal. Hence, a number of advanced BW suppression algorithms including linear low-pass filters, nonlinear filters, polynomial interpolation, wavelet filters, and mathematical morphological filters (MMF) are proposed [3–9].

Linear filters can effectively filter the high-frequency signals but cannot remove the additive noise, which has a frequency band similar to that of ECG signals. Polynomial interpolation depends on the accurate determination of knots and may be unreliable during knot separation [10]. As a nonlinear filtering technique, MMF can obtain local shape features in signals by structuring the element sequences [11, 12]. However, its applications may result in “step-like” waveform distortions. Wavelet transform (WT) has also been used in BW removal. In [3], BW is estimated from the discrete WT coefficients at level *j* and is subtracted from the original ECG signals. WT method exhibits relatively good effects for BW suppression. However, this method causes *T* waveform distortions due to the frequency overlaps between the high-scale approximate coefficients and *T* wave. The ECG signal is reconstructed by an inverse WT, and the high-scale approximate coefficients are set to zero, thereby causing the *T* wave distortion [13].

This study introduces a combined algorithm (CA) of MMF- and WT-based filtering for BW suppression. The CA can effectively preserve the outline of the BW and avoid waveform distortions caused by morphology filters, thereby obtaining an enhanced ECG quality.

The study is organized as follows.  Section 2 describes the combined filtering method, while the simulation results are provided and quantitatively analyzed in Section 3. Finally, summary and conclusions are drawn in Section 4.

## 2. The Combined Method

Considering that the main focus of this study is BW suppression, we model the contaminated ECG as the superposition of the real ECG and the BW and ignoring other types of interferences, as follows:

(1) $$\begin{array}{c} f_{\text{CECG}}\left(n\right)=f_{\text{ECG}}\left(n\right)+f_{\text{BW}}\left(n\right), \end{array}$$

where *f*_CECG_, *f*_ECG_, and *f*_BW_ are the contaminated ECG-, real ECG-, and BW-function with respect to time index *n*, respectively. All of the time functions in this study are discrete because the implementation of filtering is focused on digital processing. The BW suppression is commonly implemented by cancellation, i.e.,

(2) $$\begin{array}{c} {\hat{f}}_{\text{ECG}}\left(n\right)=f_{\text{CECG}}\left(n\right)-{\hat{f}}_{\text{BW}}\left(n\right), \end{array}$$

where the hat symbol ^ denotes the estimation of the underneath term. The output error of this cancellation procedure is given by

(3) $$\begin{array}{c} e\left(n\right)=f_{\text{ECG}}\left(n\right)-{\hat{f}}_{\text{ECG}}\left(n\right)={\hat{f}}_{\text{BW}}\left(n\right)-f_{\text{BW}}\left(n\right), \end{array}$$

which indicates that the performance of BW suppression is determined by the estimator of *f*_*BW*_, i.e., ${\hat{f}}_{\text{BW}}$. To refine the estimator ${\hat{f}}_{BW}$, we provide a staged framework which combines *N* different filtering technology. The expression of this framework is shown as follows:

(4) $$\begin{array}{c} {\hat{f}}_{\text{BW}}\left(n\right)=\left(f_{F1}\cdot f_{F2}\cdot \ldots \cdot f_{FN}\right)\left(f_{\text{CECG}}\left(n\right)\right), \end{array}$$

where *f*_*Fn*_ is the *n*th filter and operator · denotes function composition, defined as follows:

(5) $$\begin{array}{c} \left(f\cdot g\right)\left(x\right)=f\left(g\left(x\right)\right). \end{array}$$

By choosing filters carefully, the framework is capable of combining advantages of different filter implementations, and in this study, we combine the MMF-based filter and WT-based filter.

### 2.1. The Morphological Filtering

The shortcomings of the linear BW suppression methods are caused by the nonlinear nature of the contaminated ECG. Hence, nonlinear processing methods are preferred, and the MMF belongs to this category, which is capable of maintaining the shape of the input signal. The objects of the morphological operations are sets and vectors; for the clarity of descriptions, hereafter vectors are denoted by lower case boldface letters, and *ℝ* ^*N*^ denotes the real coordinate space of *N* dimensions. The reflection of a set comprised of vectors is defined as

(6) $$\begin{array}{c} A^{r}=\left\{-a\;|\;a\in A\right\}, \end{array}$$

while the translation is given by

(7) $$\begin{array}{c} {\left(A\right)}_{z}=\left\{a+z\;|\;a\in A\right\}, \end{array}$$

and hence the dilation and erosion can be expressed as follows:

(8) $$\begin{array}{c} A⊕B=\left\{z\;|\;{\left(B^{r}\right)}_{z}\cap A\neq \emptyset \right\},\\ A⊖B=\left\{z\;|\;{\left(B\right)}_{z}\subseteq A\right\}. \end{array}$$

The morphological filter is comprised of opening and closing operators, which can be expressed by dilation and erosion, as follows:

(9) $$\begin{array}{c} A◯B=\left(A⊖B\right)⊕B, \end{array}$$

(10) $$\begin{array}{c} A•B=\left(A⊕B\right)⊖B. \end{array}$$

We can apply these morphological operators to a time function by treating the *n* − *f* plane as a binary image, and the value of the pixels underneath the curve equals one. The geometric interpretation of opening in equation (9) and closing in equation (10) is sliding a given structuring element along with the signal from beneath and above, respectively. Specifically, the result of opening comprises the highest points reached by any part of the structuring element, while closing is comprised of the lowest ones. Consequently, the semantic meaning of opening/closing is peak-suppression/pit-filling. According to the model shown in equation (1), the spiky *f*_ECG_ can be seen as the noise for *f*_BW_ estimation, and intuitively the combination of opening and closing is capable of smoothing the fluctuation introduced by the *f*_ECG_. The expression of the estimator has the following form:

(11) $$\begin{array}{c} f_{\text{MMF}}\left(f_{\text{CECG}}\right)=\frac{1}{2}\left[\left(f_{\text{CECG}}◯S•S\right)+\left(f_{\text{CECG}}•S◯S\right)\right], \end{array}$$

where *S* is the structuring element. Let the output of the filter be the estimation of *f*_BW_, i.e.,

(12) $$\begin{array}{c} {\hat{f}}_{\text{BW}}=f_{\text{MMF}}\left(f_{\text{CECG}}\right), \end{array}$$

the estimation of *f*_ECG_ can be obtained by cancellation:

(13) $$\begin{array}{c} {\hat{f}}_{\text{ECG}}=f_{\text{CECG}}-{\hat{f}}_{\text{BW}}=\left(I-f_{\text{MMF}}\right)\left(f_{\text{CECG}}\right), \end{array}$$

where *I* denotes the identity operator, i.e.,

(14) $$\begin{array}{c} I\left(f\right)=f. \end{array}$$

We test the estimator and the cancellation procedure using Massachusetts Institute of Technology-Boston's Beth Israel Hospital (MIT-BIH) arrhythmia database [14] (record number 109). Shape and size of the chosen structuring element are very important. The shape of structuring element should be as similar as the filtered signal waveform needed. The ECG baseline wander is a low-frequency signal, and its shape is more approximate to the line segment. So the morphological filters are with line segments as structuring elements. And the height of linear structuring element has little effect on the results of mathematical morphological filtering. The size of structuring element directly determines whether the noise can be better removed and whether the required signal can be better retained. The width of structural element should be wider than the noise waveform removed and narrower than the signal waveform needed to be retained. If the width of the structural element is too small, the noise component cannot be eliminated well; if the width of the structural element is too large, some signals that need to be retained will be filtered. The time duration of characteristic waves of ECG is listed in Table 1.

**Figure 1:**
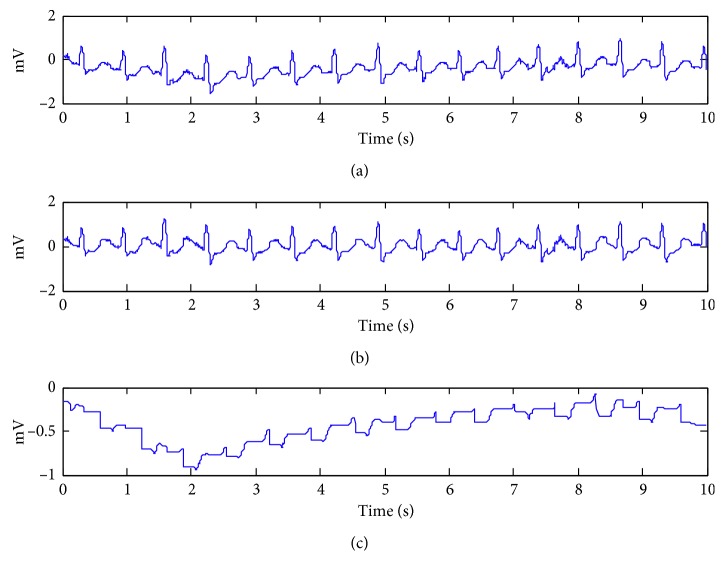
Example of removing BW using morphology filter. (a) The ECG contaminated by BW. (b) The filtered ECG signal by MMF. (c) The estimated BW.

For ECG signals with BW, the frequency range of BW noise is slightly smaller than that of *T* wave, that is, the time width of baseline drift noise waveform is larger than that of characteristic waveform of ECG signal. As mentioned above, the time width of *T* wave is 0.05–0.20 s; when the sampling rate is 360 Hz, the sampling points are 360 × 0.2=72. So, the width of structural elements selected by BW is 72 sampling points in this paper. The result is shown in Figure 1.

Theoretically, if morphological filtering with a large width of the structuring element is used to process the signal directly, the BW is obtained. Despite the high amplitude of QRS wave, the peaks and pits of its adjacent regions are also removed during the simulation, resulting in the distortion of the QRS waves and P-R segments.

### 2.2. The Combination of WT-Based Filter

Although the MMF can track the slow drifting of the baseline wandering, step-like shape shown in Figure 1(c) demonstrates that the estimated BW is still noisy. Considering various degrees of distortions, we adopt WT-based filtering to smooth the estimated BW.

Smoothing BW signal can be regarded as the elimination of high-frequency components and retention of low-frequency ones, and WT-based filtering is suitable for this kind of task. The estimated BW can be decomposed into multiple scales in the context of WT: considering the BW frequency ranges from 0.05 Hz to 2 Hz, the components below 2 Hz are preserved; while the ones above 2 Hz are replaced with zero. Finally, the smoothened BW signal can be obtained by reconstruction using the inverse wavelet transform.

Here we choose coif3 as the wavelet function because its regularity and symmetry properties are better compared with other wavelets. Also, the coif3 is the most widely used wavelet function for ECG process. For the ECG signal of which the sampling frequency is 360 Hz, the BW signal is decomposed by the WT into seven scales. After decomposition, the approximate frequency range for each scale is shown in Table 2, where *D* represents the detailed components (high-frequency components) of the signal at the scale after wavelet decomposition and A represents the approximate components (low-frequency components) at the scale. The seventh approximate component is reserved.

We denote the WT-based filter as *f*_WT_ and combine it with *f*_MMF_ according to the framework shown in equation (4); hence, the expression of the combined filter (or BW estimator) can be written as follows:

(15) $$\begin{array}{c} {\hat{f}}_{\text{BW}}=\left(f_{\text{WT}}\cdot f_{\text{MMF}}\right)\left(f_{\text{CECG}}\right), \end{array}$$

and the estimated ECG is given by the following cancellation:

(16) $$\begin{array}{c} {\hat{f}}_{\text{ECG}}=\left(I-f_{\text{WT}}\cdot f_{\text{MMF}}\right)\left(f_{\text{CECG}}\right). \end{array}$$

**Figure 2:**
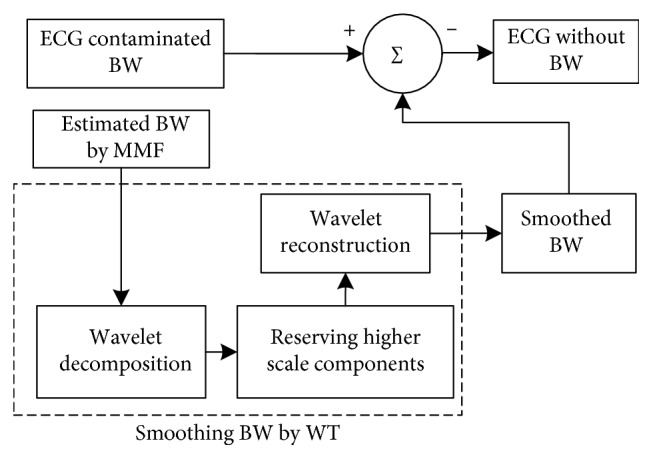
Block diagram of the CA.

The entire block diagram of the combined algorithm of the MMF and WT is shown in Figure 2.

## 3. Numerical Simulation

In real ECG recordings, the exact ECG value and BW noise are unknown which prevents one from analyzing algorithm performance with precision. Hence, a simulated ECG signal plus BW noise is used to evaluate the effectiveness of the CA proposed in this study [15].

The generation of the simulated contaminated ECG is based on equation (1), while *f*_BW_ used here is collected clinically, and the simulated *f*_ECG_ is constructed by

(17) $$\begin{array}{c} f_{\text{ECG}}\left(n\right)=\underset{m}{\sum }f_{\text{SHB}}\left(n+mT\right), \end{array}$$

where *f*_SHB_(*n*) is the waveform of a single heartbeat of which the duration is *T* and ∑_*m*_ *f*_SHB_(*n*+*mT*) is the periodic repetition of the heartbeat waveform representing a simulated clean ECG signal where *m* ∈ *ℤ* ^+^.

**Figure 3:**
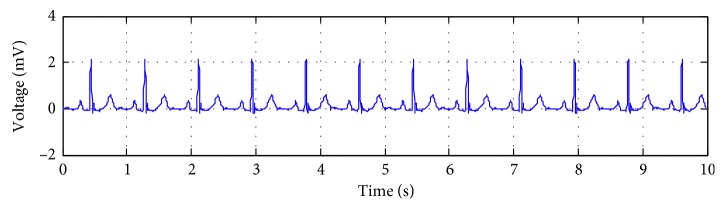
Artificial ECG signal.

**Figure 4:**
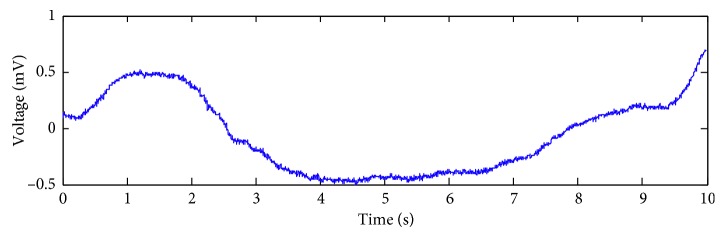
The chosen BW.

The specific process of obtaining the artificial ECG (i.e., *S*) is described as follows:

1. One heartbeat of an ECG recording, which is from the recording of number 119 in MIT-BIT arrhythmia database sampled at 360 Hz in resting conditions, is selected. The clean ECG is formed by periodic repetitions of a single beat at 1000 times. The clean ECG segment used in the experiment is subsequently obtained. An example is shown in Figure 3.
2. The second channel of the BW data from the MIT-BIH noise stress test database is selected as the BW [16].  Figure 4 shows the chosen BW signal.

The performance of the proposed method was evaluated by the mean square error (MSE) and the signal-to-noise ratio (SNR), which are defined as formulas (18) and (19), respectively.

(18) $$\begin{array}{c} \text{MSE}=\frac{1}{N}\overset{N-1}{\underset{n=0}{\sum }}{\left(f_{\text{ECG}}\left(n\right)-{\hat{f}}_{\text{ECG}}\left(n\right)\right)}^{2}, \end{array}$$

(19) $$\begin{array}{c} \text{SNR}=10\times \mathrm{lg}\left(\frac{\theta^{2}}{\text{MSE}}\right), \end{array}$$

where *θ* ^2^ is the variance of the ECG, defined as

(20) $$\begin{array}{c} \theta^{2}=\frac{1}{N}\overset{N-1}{\underset{n=0}{\sum }}{\left(f_{\text{ECG}}\left(n\right)-{\bar{f}}_{\text{ECG}}\right)}^{2}, \end{array}$$

where ${\bar{f}}_{\text{ECG}}$ is the mean of the ECG. The calculated values of the MSE and SNR for the artificial contaminated ECG signal filtered by the algorithms are listed in Table 3.

The MSE value is small, indicating a smaller error between the filtered ECG and the clean ECG. The distortion produced by the filter is also small. Meanwhile, the SNR value of the filtered ECG is high, indicating that the algorithm works for the BW interference suppression.

As can be seen from Table 2, the effect of MMF is significantly better than that of WT in the BW suppression. The reason is that the frequency range of the *T* wave in ECG signal is partially overlapped with the frequency range of BW noise. When the high-scale approximate components of wavelet decomposition are set to zero, wavelet reconstruction could cause *T* wave distortion. The CA gets the smallest MSE and highest SNR, which demonstrates that the performance of BW suppression is better.

## 4. Statistical Analysis

To further perform evaluation of CA, a statistical analysis scheme is considered [17]. Other two baseline removal algorithms used regularly in literatures, which are Butterworth high-pass filter [18] and wavelet-based high-pass filtering [19], are introduced and compared.

The original artificial ECG signals for this experiment are generated using the ECGSYM software [20] which allows configuring ECG parameters (such as heart rate, sampling frequency, the morphology of the ECG waves, amplitude and duration parameters, etc). For the experiment, four segments of artificial ECG signals with different heart rates, which are 40 beats per minute (bpm) (bradycardia), 70 bpm (normal), 90 bpm (tachycardia), and 120 bpm (exercise), are generated, respectively, and the sampling frequency is set to 360 Hz and duration of the signal to 5 min. Afterwards, the real baseline drifts from the MIT-BIH Noise Stress Test Database [21] are added to the artificial ECG.

Three performance indexes are chosen to evaluate the algorithms besides above MSE, which are described below.

### 4.1. Correlation Coefficient (CC)

It is used to quantify impairment in the morphology of the filtered signals. It is independent from scaling or offsetting the signals and focuses on the matching form of original and filtered waveforms [17]. Mathematically, the correlation coefficient between the original signal *x*(*t*) and the filtered one $\hat{x}\left(t\right)$ is given by

(21) $$\begin{array}{c} \text{CC}\left\{x\left(t\right),\hat{x}\left(t\right)\right\}=\frac{E\left[\left(x\left(t\right)-\mu_{x}\right)\left(\hat{x}\left(t\right)-\mu_{\hat{x}}\right)\right]}{\sigma_{x}\sigma_{\hat{x}}}, \end{array}$$

where *E*[·] denotes the expected value operator, *μ*_*x*_ is the expected value of *x*(*t*), and *σ*_*x*_ is its standard deviation.

### 4.2. L_Operator (LO)

It is a measurement of similarity that is based on the Euclidian distance between the two signals [21]. Mathematically, it is given by

(22) $$\begin{array}{c} \text{LO}\left\{x\left(t\right),\hat{x}\left(t\right)\right\}=1-\frac{E\left[{\left(x\left(t\right)-\hat{x}\left(t\right)\right)}^{2}\right]}{E\left[x^{2}\left(t\right)\right]+E\left[{\hat{x}}^{2}\left(t\right)\right]}. \end{array}$$

In contrast to the correlation coefficient, the LO is sensitive to offsetting and scaling of any of the two signals [17].

### 4.3. Absolute Maximum Distance (AMD)

It is one of the most commonly similarity metrics used to determine the quality of ECG signals after performing a filtering process and can be defined by the following expression [22]:

(23) $$\begin{array}{c} \text{AMD}\left\{x\left(t\right),\hat{x}\left(t\right)\right\}=\text{max}\left|x\left(t\right)-\hat{x}\left(t\right)\right|,\;\left(1\leq m\leq r\right), \end{array}$$

where *m* is the number of the current sample of the signals and *r* is the maximum number of samples of the *x*(*t*) and $\hat{x}\left(t\right)$ signals.

It allows to measure the accumulated error and gives differences in all their extension.

The average results of the comparison study are presented in Table 4.

The results demonstrated that even though there are small differences among the methods, they were all good performers in terms of CC, LO, AMD, and MSE. However, we see that the method that best maintained the original ECG morphology is CA (highest CC and LO and lowest AMD and MSE). The reason for this is probably due to the combination of MMF and WT whose features match precisely the time and frequency domain properties of the artifact.

The second best performance, according to the indexes, is yielded by the WT method. It is probably due to the properties of the chosen suited wavelet and the relatively high decomposition level. Although wavelet-based high-pass filtering method is very similar to the wavelet-based method, a high-pass filtering (an infinite impulse response filter of order one and a cutoff frequency of 0.5 Hz) is used on the approximation coefficients instead of setting them to zero. This is somewhat comparable to a soft threshold on the approximation coefficients. And the Vaidyanathan-Hoang wavelet, not coif3 used in WT, is used [17, 19].

According to the indexes, Butterworth (lowest CC and highest MSE) and MMF (lowest LO and highest AMD) show a similar worst performance. Even so, computationally Butterworth can be approximated as a finite impulse response filter, and MMF significantly reduces the amount of computation by opening and closing operators. They both have speedy computation and especially suit for medical applications that require fast but still accurate signal processing algorithms.

## 5. Conclusion

In presence of baseline wanders, there is a need to use a promising technique for baseline drifts suppression. In this paper, we have presented and validated a combined algorithm of mathematical morphology filter and wavelet transform for baseline wandered ECG signals. Compared with the current state-of-the-art methods, the filtering effect of the presented algorithm is better, and it can effectively filter out the BW in the ECG signal meanwhile keeping the distortion of the ECG signal minimized (the smallest MSE and highest SNR). This gives the opportunity to study very low amplitude complexes, and therefore, it is suited for the data preprocessing for precise ECG characteristic extraction.

## Figures and Tables

**Table 1:** Time duration of characteristic waves of ECG signal.

Characteristic waves	*P* wave	QRS wave	*T* wave
Time duration (s)	0.08∼0.11	0.06∼0.10	0.05∼0.20

**Table 2:** Frequency ranges of the estimated BW signal decomposition with seven scales.

Wavelet coefficients	Frequency ranges (Hz)
D1	90–180
D2	45–90
D3	22.5–45
D4	11.3–22.5
D5	5.6–11.3
D6	2.8–5.6
D7	1.4–2.8
A7	0–1.4

**Table 3:** Values of the MSE and SNR.

Signal	MSE	SNR
Artificial ECG	0.1170	3.0757
ECG filtered by WT	0.0173	8.9145
ECG filtered by MMF	0.0051	13.5224
ECG filtered by CA	0.0024	16.7154

**Table 4:** The average results.

Methods/indexes	CC	LO	AMD (mV)	MSE
Butterworth	0.9742	0.9718	10.2	0.0128
Wavelet high-pass	0.9801	0.9826	4.99	0.0096
WT	0.9895	0.9890	4.10	0.0072
MMF	0.9791	0.9705	15.91	0.0109
CA	0.9937	0.9929	2.59	0.0049

## Data Availability

The ECG data used to support the findings of this study have been deposited in the MIT-BIH arrhythmia database (https://doi.org/doi:10.13026/C2F305).
